# Pneumonie organisée révélatrice d'une polymyosite

**DOI:** 10.11604/pamj.2014.19.116.4939

**Published:** 2014-10-01

**Authors:** Ahmed Ben Saad, Samah Joobeur, Naceur Rouetbi, Saousen Cheikh Mhamed, Néji Skhiri, Hathami Mribah, Ali El Kamel

**Affiliations:** 1 Service de Pneumologie et d'Allergologie, Hôpital Universitaire Fattouma Bourguiba, 5000 Monastir, Tunisie

**Keywords:** Pneumonie organisée, polymyosite, connectivites

## Abstract

Les polymyosites (PM) sont des connectivites très rares, d’étiologie inconnue, dotées d'un grand polymorphisme clinique et évolutif. Elles peuvent être associées à d'autres manifestations viscérales notamment pulmonaires telles que la pneumopathie interstitielle. Ces complications respiratoires sont souvent associées à un taux de mortalité élevé. Les cas de pneumonie organisée révélatrice de polymyosite sont rarement rapportés dans la littérature et de description récente. Nous rapportons l'observation d'une patiente âgée de 53 ans qui a présenté, 14 mois après avoir porter le diagnostic d'une pneumonie organisée, des myalgies diffuses, un ‘dème des membres inférieurs et une élévation des enzymes musculaires. La biopsie musculaire a confirmé le diagnostic de la myosite. L’évolution était favorable sous corticothérapie. Le traitement de la PO associée au PM n'est pas clairement établi. La corticothérapie constitue le traitement de première intention.

## Introduction

La polymyosite (PM) est une maladie inflammatoire chronique, rare, pouvant donner des manifestations viscérales, notamment pulmonaires. L'association pneumonie organisée (PO) polymyosite est de description récente. Nous rapportons un cas de polymyosite révélée par une pneumonie organisée.

## Patient et observation

**Figure 1:**
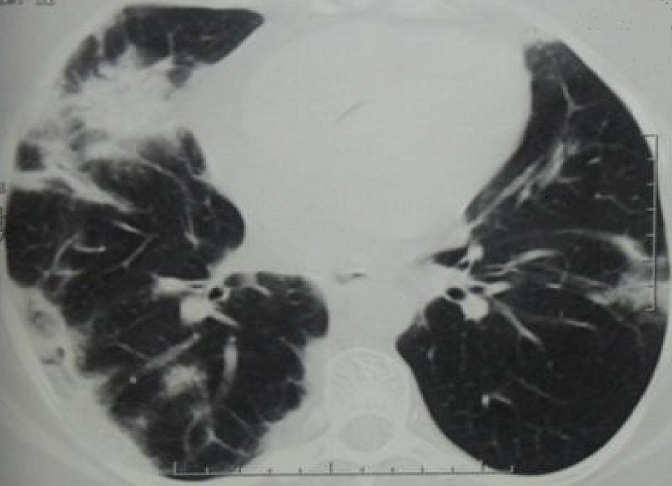
Scanner thoracique: multiples foyers de condensation alvéolaire à disposition périphérique évoquant une PO

**Figure 2:**
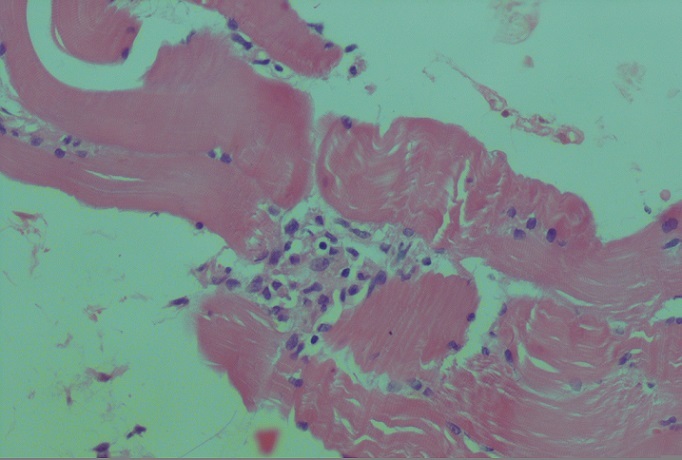
Biopsie musculaire (HEx100): infiltrat lymphocytaire endomysial léger

Il s'agit d'une patiente âgée de 53 ans, qui était admise pour dyspnée d'effort d'aggravation récente avec arthralgies des grosses articulations. A l'examen clinique, on notait la présence de râles crépitants à l'auscultation pulmonaire. La radiographie du thorax montrait des opacités confluentes floues prédominant aux bases. Le scanner thoracique avait objectivé de multiples foyers de condensation alvéolaire à disposition périphérique évoquant une PO (Figure 1). Le bilan fonctionnel avait conclu à un trouble ventilatoire restrictif (CVF: 1.67L soit 69% de la valeur prédite, VEMS: 1.4L soit 69% de la prédite et un rapport VEMS/CV à 84%) avec baisse de la DLCO à 40% et une hypoxie (PaO2 à 67 mmHg). L'enquête étiologique initiale était revenue négative (Pas de prise médicamenteuse, bilan immunologique et infectieux négatifs) à part le dosage du facteur rhumatoïde qui était revenu positif avec Anti CCP négatif. Elle avait bénéficié d'une corticothérapie à base de prednisone à la dose initiale de 1 mg/Kg/j. L’évolution au bout de 2 mois était marquée par l'amélioration clinique, fonctionnelle respiratoire (CVF: 2.19L soit 90% de la prédite, VEMS: 1.65L soit 80% de la prédite, VEMS/CV: 75%, DLCO: 66% et PaO2: 80.5 mmHg) et radiologique sans nettoyage total. Le scanner thoracique de control avait objectivé des images de condensation alvéolaires des deux bases contenant des bronchectasies avec épaississement des septas périlobulaires: aspect en faveur de PO avec fibrose débutante. Quatorze mois après le début des manifestations respiratoires, installation de myalgies diffuses, impotence fonctionnelle des épaules, œdème des membres inférieurs avec élévation des enzymes musculaires. La biopsie musculaire avait montré la présence de cellules musculaires nécrosées et entourées par un infiltrat inflammatoire fait par des histiocytes et par des lymphocytes; cet infiltrat était visible par endroits autour des cellules musculaires striées d'aspect normal (Figure 2). Ces constatations anatomopathologiques ont permis de conclure à une myosite. La patiente a été traitée par bolus de solumédrol puis par prednisone (1.5 mg/Kg) avec amélioration clinique et biologique de sa PM. Par ailleurs, elle était restée stable sur le plan fonctionnel respiratoire et radiologique.

## Discussion

Les polymyosites (PM) et dermatomyosites (DM) sont des connectivités très rares, d’étiologie inconnue, dotées d'un grand polymorphisme clinique et évolutif. Elles appartiennent au groupe de myopathies inflammatoires idiopathiques caractérisées par une inflammation chronique des muscles striés [1]. Si les PM et les DM intéressent, par définition, les muscles striés et la peau, elles sont également associées à d'autres manifestations viscérales [1, 2]. Les complications pulmonaires au cours des PM surviennent chez 5 à 45% des patients [1, 3], et elles sont essentiellement représentées par la pneumopathie interstitielle, les pneumonies d'inhalation dues en partie aux troubles moteurs pharyngo-œsophagiens, et l'hypoventilation alvéolaire secondaire à une atteinte des muscles respiratoires (paralysie diaphragmatique, déficit des muscles intercostaux et respiratoires accessoires) [4, 5, 6]. Ces complications respiratoires sont souvent associées à un taux de mortalité élevé [5]. La pneumopathie interstitielle non spécifique représente la forme histopathologique le plus souvent retrouvée au cours des PM (40 à 80%) [2, 7, 8]. La PO est rarement associée à la PM [8, 9]. Cette association est récemment rapportée [7, 10]. La PO représente 4 à 38% des cas d'atteinte interstitielle pulmonaire associés à la PM/DM selon les séries [2]. Sa date de survenue est variable dans le cours évolutif des PM [2]. La PO peut se manifester par une dyspnée à l'effort, accompagnée ou non de toux sèche ou bien rester complètement asymptomatique [2, 7, 11]. Il n'y'a pas de particularités cliniques ou épidémiologiques de la PO en cas d'association à la DM [10] et il n'y'a pas de corrélation entre la sévérité de l'atteinte musculaire de la PM et respiratoire de la PO [11].

La tomodensitométrie haute résolution (TDM-HR) est l'examen radiologique de choix [2]. Elle fournit des informations concernant l'activité, l'ancienneté, la sévérité et oriente sur le type d'atteinte histologique de la PID [2]. La présence de zones de condensation sous-pleurales et d'opacités linéaires (épaississements septaux) traduirait une PO. Ces anomalies siègent préférentiellement dans les lobes inférieurs et les régions postérieures [2]. Les explorations fonctionnelles respiratoires objectivent un syndrome restrictif avec une réduction de la capacité vitale forcée, de la capacité pulmonaire totale et de la DLCO d'intensité variable [2]. La diminution de la DLCO est l'anomalie la plus précoce au cours des pneumopathies interstitielles des polymyosites/dermatomyosites [3, 12]. La gazométrie artérielle montre une hypoxie et une hypocapnie ainsi qu'une élévation du gradient alvéolocapillaire pour l'oxygène à l'effort, puis au repos [2, 11].

L’étude histopathologique fournit les caractéristiques de la PO. Elle est déterminée par la présence de tissu de granulation fibreux, constitué d'infiltrats de cellules inflammatoires non spécifiques (lymphocytes, macrophages, polynucléaires neutrophiles et fibroblastes), et de tissu conjonctif, aboutissant à l'obstruction endoluminale des espaces aériens distaux (bronchioles terminales, canaux alvéolaires et alvéoles). L'architecture pulmonaire est conservée [2]. Le lavage broncho-alvéolaire montre une prédominance des neutrophiles et l'inversion du rapport CD4+/CD8+ [13].

Le traitement de la PO associée au PM n'est pas clairement établi [2, 8]. La corticothérapie constitue le traitement de première intention à la dose initiale quotidienne de 1 mg/kg d’équivalent prednisone, avec dégression progressive [3, 7, 13]. L'absence de réponse aux glucocorticoïdes ou l'apparition des effets secondaires rend nécessaire l'introduction d'un traitement immunosuppresseur (IS) [13]. La corticorésistance au cours des PO est observée plus avec les DM que les PM [10]. L'atteinte interstitielle pulmonaire au cours des PM est considérée comme un facteur de mauvais pronostic car souvent responsable d'une lourde mortalité par insuffisance respiratoire [2, 5]. Cependant le pronostic est meilleur avec la PO qu'en cas d'association avec une autre atteinte interstitielle (pneumopathie interstitielle usuelle ou le dommage alvéolaire diffus) [2, 10, 11].

## Conclusion

Les complications pulmonaires au cours des PM surviennent chez 5 à 45% des patients [3, 5] et elles sont essentiellement représentées par la pneumopathie interstitielle. L'atteinte respiratoire peut précéder les manifestations musculaires. La pneumonie organisée est observée dans 4 à 38% des cas d'atteintes interstitielles pulmonaires associées à la PM. Il n'y a pas de particularités cliniques de la PO en cas d'association à une PM. Le traitement de première intention est à base de corticoïde avec évolution favorable dans la majorité des cas. Les limites de données concernant cette association serait en rapport avec la rareté de l'association d'une part, et probablement l'absence de confirmation histopathologique de la PO dans certains cas.
